# Supplementary material for: Genome-Wide Association Study of Body Conformation Traits in a Three-Way Crossbred Commercial Pig Population
Source: Animals (Basel). 2023 Jul 26;13(15):2414. doi: 10.3390/ani13152414 (PMC10417164; doi:10.3390/ani13152414)
Supplement: Supplementary file 1 [file animals-13-02414-s001.zip › animals-2440871-supplementary.pdf]

Supplementary Material:

Table S1: GO and KEGG enrichment results.

Trait	Pathway	Database	ID	Count	*P* Value
BL	ion transmembrane transport	Gene Ontology	GO:0034220	4	7.86E-03
	neuron projection	Gene Ontology	GO:0043005	5	1.17E-02
	nervous system process	Gene Ontology	GO:0050877	3	1.17E-02
	transmembrane receptor protein tyrosine kinase activity	Gene Ontology	GO:0004714	3	1.17E-02
	regulation of dendritic spine morphogenesis	Gene Ontology	GO:0061001	2	1.17E-02
	dopaminergic synapse	Gene Ontology	GO:0098691	2	1.17E-02
	integral component of postsynaptic membrane	Gene Ontology	GO:0099055	2	2.16E-02
	mechanosensitive ion channel activity	Gene Ontology	GO:0008381	2	2.37E-02
	neurotransmitter receptor activity	Gene Ontology	GO:0030594	3	2.37E-02
	regulation of membrane potential	Gene Ontology	GO:0042391	3	2.37E-02
	acetylcholine-gated cation-selective channel activity	Gene Ontology	GO:0022848	2	2.38E-02
	peptidyl-tyrosine phosphorylation	Gene Ontology	GO:0018108	3	2.58E-02
	integral component of plasma membrane	Gene Ontology	GO:0005887	7	2.64E-02
	transmembrane receptor protein tyrosine kinase signaling pathway	Gene Ontology	GO:0007169	3	2.64E-02
	motor neuron axon guidance	Gene Ontology	GO:0008045	2	2.76E-02
	integral component of presynaptic membrane	Gene Ontology	GO:0099056	2	2.76E-02
	hemopoiesis	Gene Ontology	GO:0030097	2	3.43E-02
	regulation of mitotic cell cycle	Gene Ontology	GO:0007346	2	3.43E-02
	positive regulation of protein tyrosine kinase activity	Gene Ontology	GO:0061098	2	3.77E-02
	regulation of synaptic vesicle exocytosis	Gene Ontology	GO:2000300	2	4.11E-02
	endosome to lysosome transport	Gene Ontology	GO:0008333	2	4.18E-02
	chemical synaptic transmission	Gene Ontology	GO:0007268	3	4.95E-02
CC	C-C chemokine receptor activity	Gene Ontology	GO:0016493	3	1.75E-04
	chemotaxis	Gene Ontology	GO:0006935	3	4.02E-04
	chemokine-mediated signaling pathway	Gene Ontology	GO:0070098	3	8.70E-04
	C-C chemokine binding	Gene Ontology	GO:0019957	2	5.03E-03
	chemokine binding	Gene Ontology	GO:0019956	2	5.03E-03
	endoplasmic reticulum organization	Gene Ontology	GO:0007029	2	6.20E-03
	immune response	Gene Ontology	GO:0006955	3	6.20E-03
	negative regulation of apoptotic process	Gene Ontology	GO:0043066	3	1.72E-02
	cell chemotaxis	Gene Ontology	GO:0060326	2	1.72E-02
	calcium-mediated signaling	Gene Ontology	GO:0019722	2	1.72E-02
	positive regulation of cytosolic calcium ion concentration	Gene Ontology	GO:0007204	2	1.95E-02
	Golgi organization	Gene Ontology	GO:0007030	2	2.59E-02
	Viral protein interaction with cytokine and cytokine receptor	KEGG PATHWAY	ssc04061	2	3.59E-02
	Spliceosome	KEGG PATHWAY	ssc03040	2	3.72E-02

	positive regulation of chondrocyte proliferation	Gene Ontology	GO:1902732	1	3.72E-02
	plus-end-directed vesicle transport along microtubule	Gene Ontology	GO:0072383	1	3.72E-02
	negative regulation of fibroblast apoptotic process	Gene Ontology	GO:2000270	1	3.72E-02
	negative regulation by host of viral process	Gene Ontology	GO:0044793	1	3.72E-02
	negative regulation of lipopolysaccharide-mediated signaling pathway	Gene Ontology	GO:0031665	1	3.72E-02
	negative regulation of ATPase activity	Gene Ontology	GO:0032780	1	3.72E-02
	endoplasmic reticulum tubular network membrane	Gene Ontology	GO:0098826	1	3.72E-02
	COPII vesicle coating	Gene Ontology	GO:0048208	1	3.72E-02
AC	actin filament network formation	Gene Ontology	GO:0051639	2	2.60E-02
	urate metabolic process	Gene Ontology	GO:0046415	2	2.60E-02
	actin filament severing	Gene Ontology	GO:0051014	2	2.60E-02
	positive regulation of filopodium assembly	Gene Ontology	GO:0051491	2	3.81E-02
	sarcomere organization	Gene Ontology	GO:0045214	2	4.58E-02
WC	carbohydrate binding	Gene Ontology	GO:0030246	5	9.75E-03
	heart morphogenesis	Gene Ontology	GO:0003007	3	1.38E-02
	threonine catabolic process	Gene Ontology	GO:0006567	2	3.33E-02
	activation of phospholipase C activity	Gene Ontology	GO:0007202	2	4.25E-02
	DNA demethylation	Gene Ontology	GO:0080111	2	4.25E-02

Figure S1: Five trait phenotype normal distribution density map

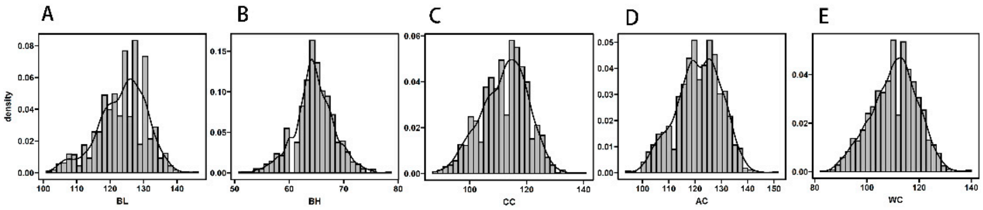

Figure S2: Q–Q plot of single-locus GWAS for five conformation traits in DLY.

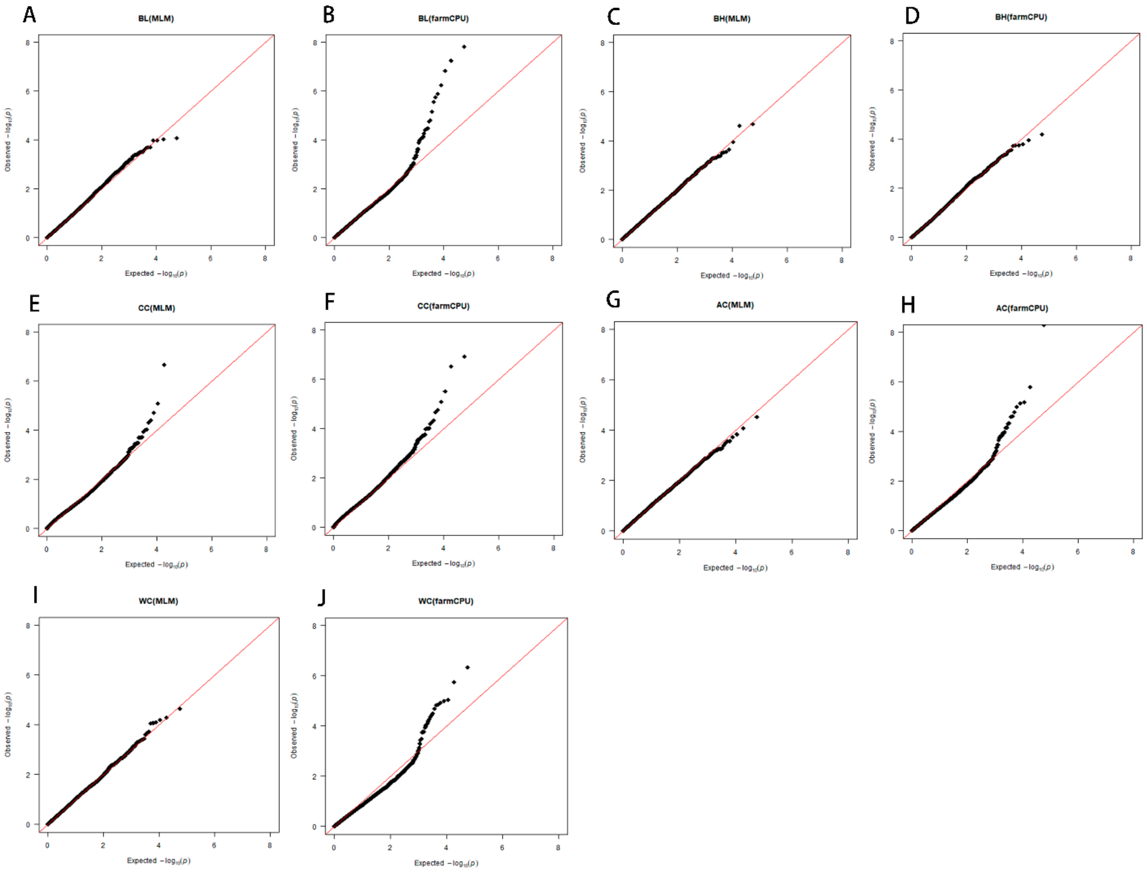

The Q–Q plot is plotted with the x- axis representing the actual measured value of −log10 (P-value) and the y-axis representing the observed value of −log10 (P-value).
